# Cutaneous allodynia as predictor for treatment response in chronic migraine: a cohort study

**DOI:** 10.1186/s10194-023-01651-9

**Published:** 2023-08-30

**Authors:** Judith A. Pijpers, Dennis A. Kies, Erik W. van Zwet, Irene de Boer, Gisela M. Terwindt

**Affiliations:** 1 Department of Neurology, Leiden University Medical Centre, PO Box 9600, 2300 RC Leiden, The Netherlands; 2 Department Radiology, Leiden University Medical Centre, Leiden, The Netherlands; 3 Department Medical Statistics, Leiden University Medical Centre, Leiden, The Netherlands

**Keywords:** Chronic migraine, Medication overuse, Allodynia, Central sensitization, Biomarker

## Abstract

**Background:**

Central sensitisation is an important mechanism in migraine chronification. It is presumed to occur in second and third order neurons sequentially, resulting in an analogous spatial distribution of cutaneous allodynia with cephalic and extracephalic symptoms. We investigated whether allodynia, and its subtypes based on spatial distribution and type of stimulus, predict response to treatment in chronic migraine patients.

**Methods:**

This study was conducted as part of the CHARM study (NTR3440), a randomized, double-blind, placebo-controlled trial in chronic migraine patients with medication overuse. We included 173 patients. The presence of cutaneous allodynia at baseline was established with the Allodynia Symptom Checklist. Primary endpoint was reversion from chronic to episodic migraine.

**Results:**

Of all patients, 74.6% reported cutaneous allodynia. Absence of allodynia compared to presence of allodynia was predictive for reversion from chronic to episodic migraine, odds ratio (OR): 2.45 (95% CI: 1.03–5.84), *p* = 0.042. The predictive value was more pronounced when subdivided for spatial distribution, for participants without allodynia versus cephalic (OR: 4.16 (95% CI: 1.21–14.30), *p* = 0.024) and extracephalic (OR: 7.32 (95% CI: 1.98- 27.11), *p* = 0.003) allodynia. Mechanical, but not thermal, allodynia, was associated with outcome.

**Conclusions:**

Cutaneous allodynia, an important marker for central sensitization, likely has predictive value for treatment response in chronic migraine.

**Graphical Abstract:**

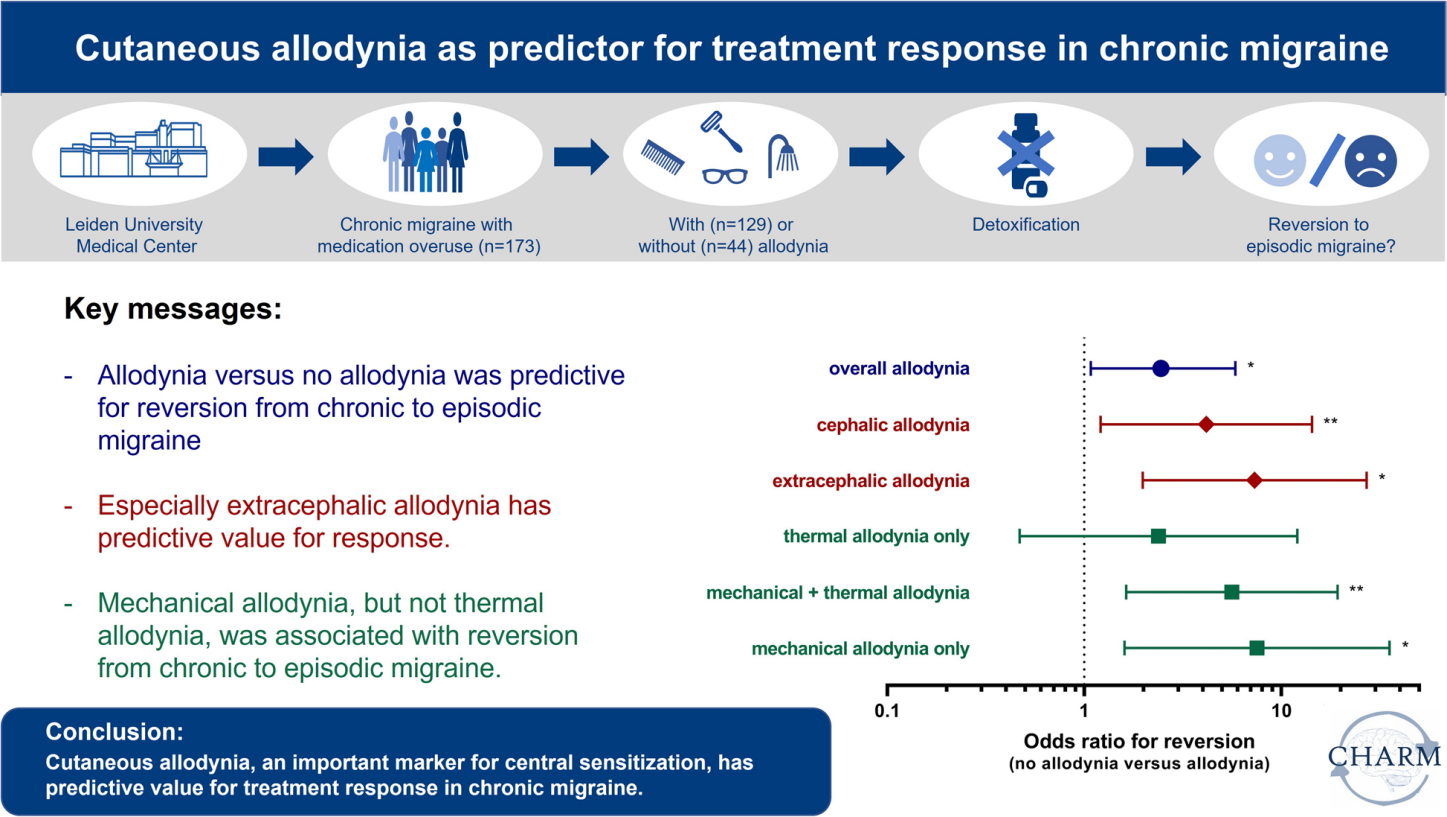

**Supplementary Information:**

The online version contains supplementary material available at 10.1186/s10194-023-01651-9.

## Introduction

Migraine is a common, multifactorial brain disorder, characterized by recurrent headache attacks with nausea, vomiting and hypersensitivity to movement, light and sound, and sometimes with aura symptoms. Most patients have the episodic form, with a median attack frequency of 1–2 per month [1]. However, every year 3% of these patients convert from less-frequent episodic migraine to high-frequent chronic migraine (≥ 15 headache days per month, of which ≥ 8 migraine days), a process called migraine chronification [2, 3]. Frequent use of acute headache medication is a major risk factor for migraine chronification, and as such, the majority of chronic migraine patients have medication overuse. Discontinuation of the overused medication is an important therapy, which is effective in the majority, but not in all patients [3, 4].

Migraine chronification is hypothesised to be a decreased threshold problem, in which patients have increased susceptibility for migraine attacks. This increased susceptibility may be a consequence of central sensitisation, a state of ongoing excitability and hyper-responsiveness of central regions of the brain [3, 5–7]. During the headache phase of a migraine attack, the trigeminal afferents surrounding meningeal blood vessel become activated [5, 8]. Recurrent activation of these trigeminal afferents induces sensitisation of the trigeminal nucleus caudalis. Due to convergence of sensory input from both the dura and the periorbital skin, sensitisation of the trigeminal nucleus caudalis results into referred ipsilateral cephalic cutaneous allodynia, i.e., the perception of pain due to a normally non-painful stimulus. Subsequently, thalamic neurons become sensitised, leading to referred extended cephalic and extracephalic cutaneous allodynia (also known as body allodynia), as all sensory input from the skin converges in the thalamus [5–7].

Thus, cutaneous allodynia, and especially the spatial distribution of cutaneous allodynia, may be used as a clinical marker of the presumably sequential central sensitisation processes. Cutaneous allodynia can be perceived upon thermal or mechanical stimuli. Hence, it is experienced during daily activities, such as combing hear, exposure to cold, wearing tight clothes, and resting the head on a pillow [9–12]. Cutaneous allodynia is associated with a higher prevalence of depression in migraine patients [13], and is an (independent) predictor for migraine chronification [9]. Preclinical and clinical studies suggests that central sensitization of the trigeminal nociceptive system is a reversible phenomenon in medication overuse [4, 14, 15]. However, few studies have evaluated cutaneous allodynia as a predictor for treatment response in chronic migraine patients. Moreover, spatial distribution of cutaneous allodynia has never been studied in the light of migraine chronification and its reversibility or as predictor of response. Therefore, the aim of this study was to investigate the association between cutaneous allodynia and its subtypes (based on spatial distribution and type of stimulus) to response to treatment in patients with chronic migraine with medication overuse.

## Material and methods

### Study design and population

This study was conducted as part of the Chronification and reversibility of migraine (CHARM) study at the outpatient headache clinic of Leiden University Medical Centre, the Netherlands, which is described in detail elsewhere [16]. Briefly, the CHARM study was a randomized, double-blind, placebo-controlled, clinical trial. Participants were enrolled between December 2012 and February 2015. While utilising data gathered from the CHARM cohort, this study focussed on the predictive value of allodynia and is therefore separate from the clinical trial that aimed to investigate whether treatment with botulinum toxin A was of added value un top of withdrawal therapy in chronic migraine patients with medication overuse headache. Consecutive patients aged 18–65 years, diagnosed with chronic migraine and medication overuse according to the formerly International Classification of Headache Disorders (ICHD) 3-beta criteria, but also fulfilling ICHD 3 criteria [2], who provided written informed consent, were enrolled. Diagnoses were made in consultation with headache experts and confirmed by a headache diary. Exclusion criteria were: (i) other primary headache or neurological disorders; (ii) other chronic pain disorders with medium to high pain intensity or requiring pain medication; (iii) major psychiatric disorders, other than depression; (iv) major cognitive, behavioural or oncologic disorders; (v) contraindications for treatment, or inability to adhere to the study protocol (vi) (planned) pregnancy or breastfeeding (vii) use of ergots, opioids or barbiturates; (viii) abuse of drugs in the past 12 months.

All participants started with a 4-week baseline-assessment period, followed by a 12-week withdrawal period, consisting of instruction to withdraw abruptly from all acute headache medications and caffeine (‘advice-only’). Prophylactic treatment was tapered off and rescue medication was not allowed. In addition to initiation of withdrawal treatment, immediately prior to withdrawal, botulinum toxin A (BTA) or placebo injections were administered in a 1:1 randomised, double-blind manner [16]. BTA and placebo were administered at 31 predefined injection sites. BTA was administered with 5 units per injection; including 155 units in total. For placebo, the 24 injections outside the forehead region contained saline and the seven injections in the forehead contained low dose BTA (2.5 units per injection site; 17.5 units in total). Moreover, participants were made clear that changes in facial expression were not indicative of any specific treatment ensuring that active treatment and placebo were not distinguishable. This insured that both participants and investigators were blinded for treatment.

The study was performed in accordance with the declaration of Helsinki Ethical Principles and Good Clinical Practices and was approved by the local and national ethics committees.

### Measurements and outcomes

All participants prospectively kept a 4-week diary, with daily registration of headache characteristics, accompanying symptoms and use of acute headache medication, during the baseline assessment period and the post treatment period (weeks 9–12). The diaries had to be sent in every week to ensure accuracy. Data (entry) was cross checked both manually (randomly) and electronically with fixed algorithms. Determination of migraine and non-migraine headache on any day was calculated by an algorithm based on the International Classification of Headache Disorders criteria. In addition, questionnaires were filled out at baseline regarding allodynia, depression and anxiety. Allodynia was questioned by the previously used and published Dutch Allodynia Symptom Checklist (ASC) [9], which is analogous to the validated English ASC [10, 17]. The ASC comprises 12 symptoms of cutaneous allodynia, namely pain or unpleasant sensation on the skin during: i) combing the hair; ii) wearing a pony tail; iii) shaving the face; iv) wearing eyeglasses; v) wearing contact lenses; vi) wearing earrings; vii) wearing a necklace; viii) wearing tight cloths; ix) taking a shower; x) resting the head on a pillow; xi) exposure to heat and xii) exposure to cold. Allodynia was scored as present when at least two of these symptoms occurred [9, 10]. To distinguish subtypes of allodynia, the 12 items were recoded based on i) spatial distribution and ii) type of stimulus. Based on the spatial distribution of referred hypersensitivity, allodynia was scored as cephalic allodynia (presence of allodynia whilst combing the hair, wearing a pony tail, shaving the face, wearing eyeglasses, wearing contact lenses, wearing earrings, taking a shower, resting the head on a pillow, exposure to heat, or exposure to cold) or extracephalic allodynia (presence of allodynia whilst wearing a necklace or wearing tight cloths). In case of both cephalic and extracephalic allodynia, the complaints were categorised as extracephalic allodynia, as extracephalic (thalamic, third order sensitisation) can be considered as more severe or advanced than cephalic (trigeminal nucleus caudalis, second order sensitisation). Based on previously performed factor analysis [9], the items were recoded based on type of stimulus as thermal (presence of allodynia whilst exposure to heat, exposure to cold or resting the head on a pillow), mechanical (presence of allodynia whilst combing the hair, wearing a pony tail, shaving the face, wearing eyeglasses, wearing contact lenses, wearing earrings, wearing a necklace, wearing tight cloths or taking a shower) or both thermal and mechanical. For the recoding into subtypes, ‘no allodynia’ was defined as absence of any allodynia symptoms. Hence, presence of cephalic, extracephalic, mechanical or thermal was scored as positive if one or more symptoms were reported, and was thus less strict compared with the overall allodynia definition, as the items per subgroup are more limited. Anxiety and depression were scored as present using a cut-off score of at least eight on the subscales of the Hospital Anxiety and Depression scale (HADS-A and HADS-D) [18].

Primary outcome was reversion from chronic to episodic migraine (i.e., headache no longer fulfils criteria of chronic migraine) from baseline to the last 4 weeks of the treatment period (weeks 9–12). Secondary outcomes were i) ≥ 50% response in migraine days, i.e., reduction in monthly migraine days (MMD) of 50% or more; ii) reduction in number of monthly migraine days (MMD); iii) reduction in number of monthly headache days (MHD). A migraine day was defined as a day fulfilling criteria for migraine with or without aura, or treated with migraine specific acute medication [2]. A headache day was defined as a day with migraine or non-migraine headache of any duration.

### Data analysis and statistics

Descriptives are reported as means ± standard deviations or numbers with proportions, and differences between groups were tested with independent sample t-tests and χ^2^ tests. Multivariate regression models were used to test the association between presence of (subtypes of) cutaneous allodynia and reversion from chronic to episodic migraine (primary endpoint), a 50% or greater reduction in migraine days, reduction in number of MMD and reduction in number of MHD (secondary endpoints). Gender, age, depression and anxiety were included in the model. Medication intake and migraine or headache days at baseline were added to the model in separate supplementary analyses, since these factors are likely related to cutaneous allodynia and the outcomes, but the magnitude and direction of these influences are not yet established. As a secondary analysis we included treatment with botulinum toxin A or placebo as a covariate. This factor was extensively tested previously, and botulinum toxin A did not significantly improve any of the outcome measures [16]. Moreover, as treatment with BTA or placebo was allocated randomly no relationship between BTA treatment and allodynia should exist. As such, we did not add this factor to our primary analyses. However, to be completely certain that the use of BTA did not influence our results we included BTA vs placebo as a covariate in our secondary analyses.

Primary analysis included all patients providing baseline data (*n* = 173). Missing data on migraine days or headache days during follow-up, defined as less than 14 completed days on a headache diary, were handled using multiple imputation. In case of 14–27 completed days, the existing data were extrapolated to a 28 days period. In all analyses, two-sided p values < 0.05 were considered statistically significant. Analyses were performed in SPSS Statistics 23.0 (SPSS Inc., ICM, USA).

### Data availability

The data that support the findings of this study are available from the corresponding author, upon reasonable request.

## Results

**Fig. 1:**
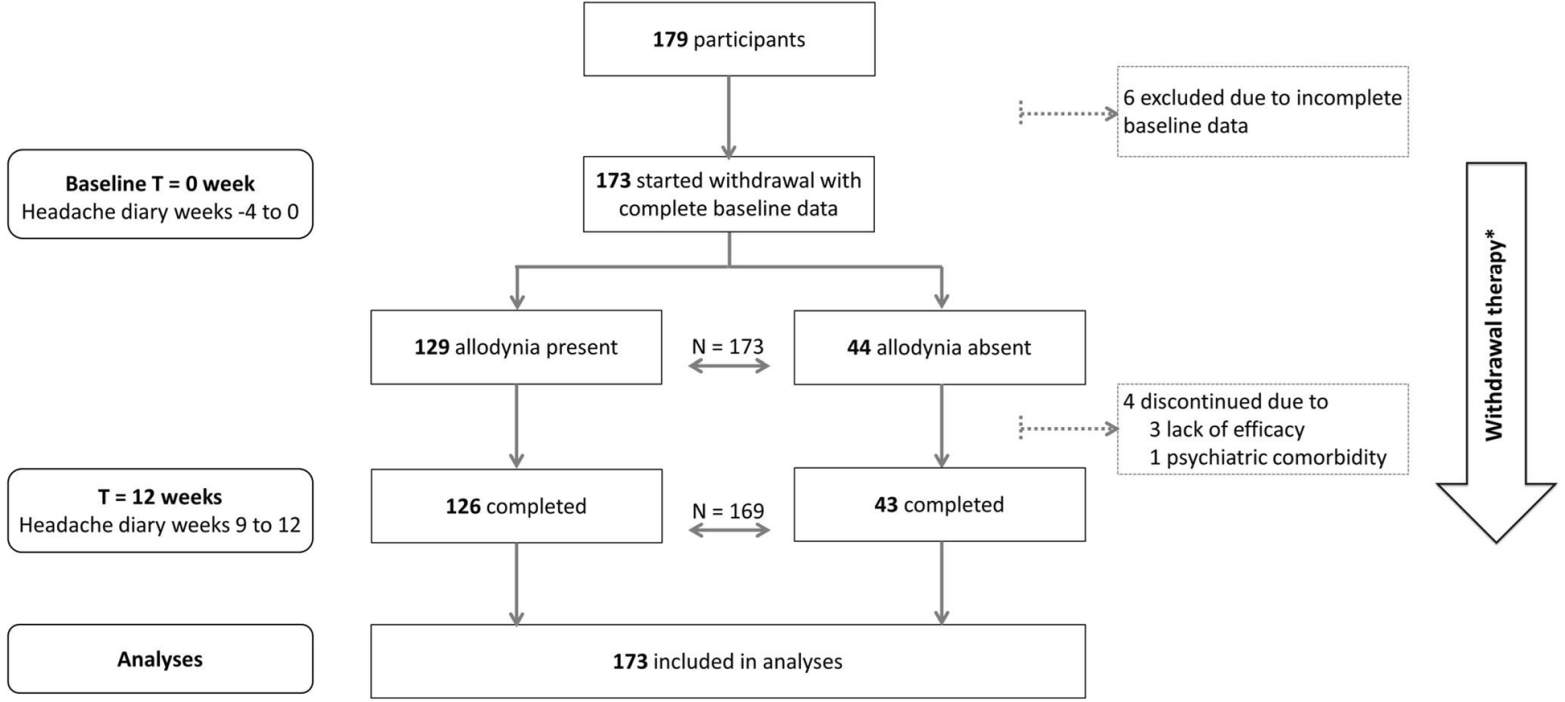
Flowchart study population. *All patients started withdrawal treatment from acute migraine mediation. Half of the patients were additionally treated with botulinum toxin A injections prior to start withdrawal in a randomized blinded fashion

The study flow is shown in Fig. 1. Of 179 participants in the CHARM study, 173 provided baseline allodynia data and were included in this current study. Of these participants, 74.6% experienced cutaneous allodynia. Almost all patients withdrew successfully (less than 2 days acute medication use per month) from acute medication (96.8% in allodynia group and 100.0% in the group without allodynia). Participants with cutaneous allodynia were mainly female and reported more often current anxiety symptomology, but did not differ on age, number of monthly migraine or headache days, age of onset, use of acute or prophylactic treatment, being treated with BTA or current depressive symptomatology (Table 1). Of all participants, 27 (16%) did not experience any allodynia symptom at all, 79 (46%) experienced at least one cephalic allodynia symptom, and 67 (38%) experienced at least one extracephalic allodynia symptom. Almost all participants who experienced extracephalic symptoms, also experienced cephalic symptoms (65 (97%)). Divided into type of stimulus, 16 participants (9%) experienced only thermal allodynia symptoms, 16 (9%) only mechanical allodynia symptoms, and 114 (66%) both thermal and mechanical allodynia symptoms.

**Table 1:** Baseline characteristics

**Variable**	**Allodynia** (*n* = 129)	**No allodynia** (*n* = 44)	*P*-value
Female	110 (85.3%)	22 (50.0%)	** < 0.001**
Age (years)	44.3 ± 10.5	47.3 ± 11.2	0.120
Age at onset	17.4 ± 9.5	17.7 ± 9.2	0.858
Monthly Migraine days (MMD)	14.9 ± 5.3	15.9 ± 6.1	0.311
Monthly Headache days (MHD)	21.5 ± 4.7	21.1 ± 5.0	0.661
Days with use of acute headache medication^a^	16.1 ± 5.4	17.1 ± 6.0	0.306
Days with use of triptans	11.1 ± 5.7	12.0 ± 7.5	0.391
Prophylactic treatment ^b^			
Current use	50 (38.8%)	13 (29.5%)	0.273
History of use	115 (89.1%)	43 (97.7%)	0.081
BTA injections prior withdrawal	61 (47.2%)	26 (59.1%)	0.222
Depression, % present (HADS-D ≥ 8)	51 (39.5%)	15 (34.1%)	0.521
Anxiety, % present (HADS-A ≥ 8)	50 (38.8%)	5 (11.4%)	**0.001**

Values are means ± SD or n (%). BTA: botulinum toxin A

^a^Any headache medication: Simple analgesics (paracetamol, NSAID’s) triptans and/or combination drugs

^b^Commonly used prophylaxis for migraine, such as beta-blockers, valproic acid or topiramate

**Fig. 2:**
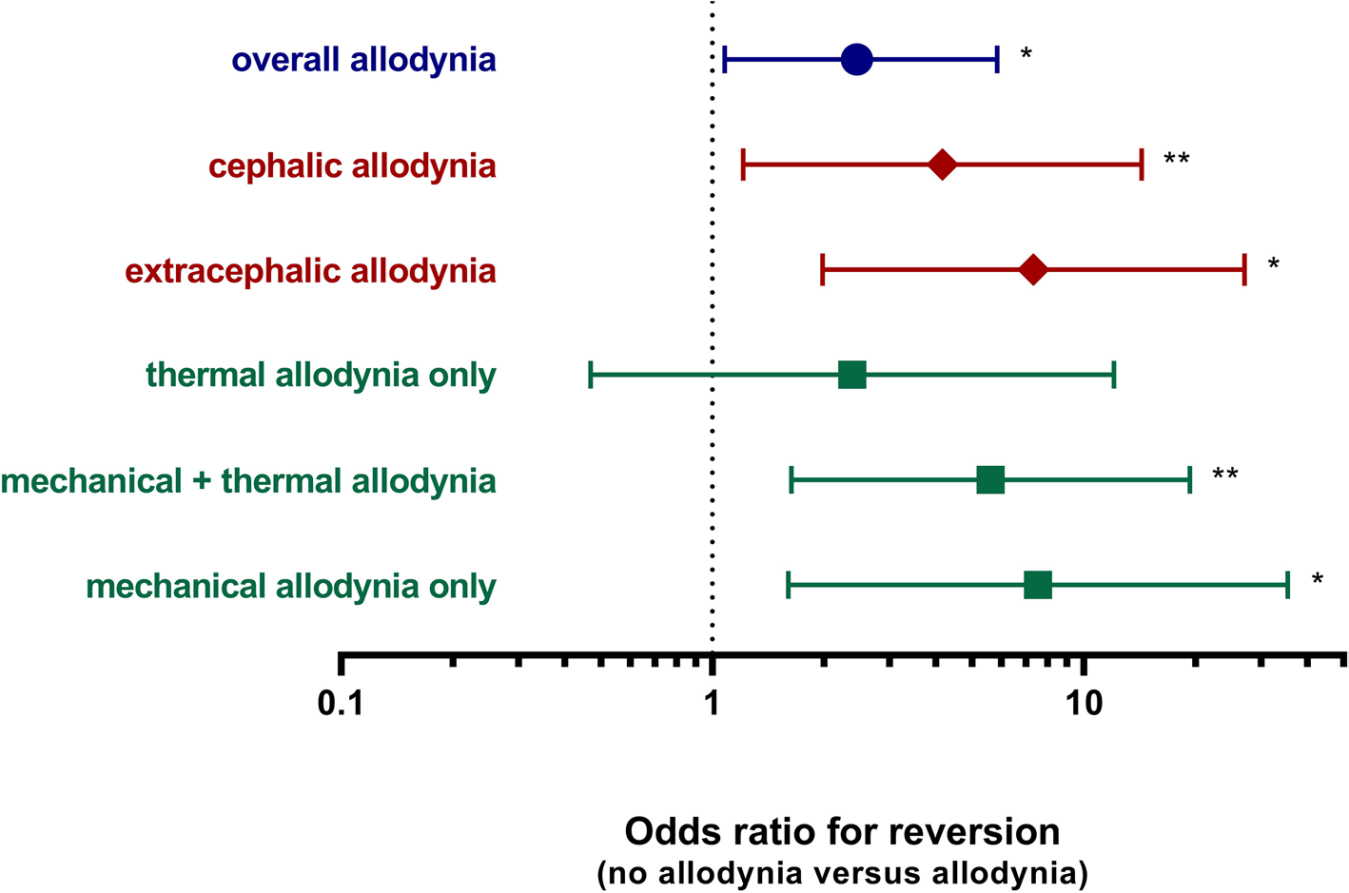
Odds ratio for reversion from chronic to episodic migraine of no allodynia compared to different subtypes of allodynia. * *p* < 0.05; ** *p* < 0.01

The absence of cutaneous allodynia was predictive for good outcome after 12 weeks. For the primary endpoint, the odds for reversion from chronic migraine to episodic migraine was 2.5 times higher for participants without allodynia compared to participants with allodynia (OR 2.45; 95% CI 1.03 to 5.84; *p* = 0.042, Table 2 and Fig. 2), as 75.0% of participants without allodynia versus 57.4% of participants with allodynia reverted to episodic migraine. The predictive value was more pronounced when allodynia was specified according to spatial distribution, with a 4 and 7 times higher odds for reversion to episodic migraine for participants without allodynia compared to participants with cephalic allodynia and extracephalic allodynia respectively (no allodynia versus cephalic allodynia OR 4.16; 95% CI 1.21 to 14.30; *p* = 0.024, no allodynia versus extracephalic allodynia OR 7.32; 95% CI 1.98 to 27.11, *p* = 0.003). When subdivided by type of stimulus, both the combination of mechanical plus thermal allodynia and mechanical allodynia alone were predictive for reversion to episodic migraine, whereas thermal allodynia alone was not predictive (Table 2 and Fig. 2). See Table S1 for the percentage of participants that reached the outcome (conversion CM to EM and 50% reduction MMD) in the different subgroups.

**Table 2:** Allodynia as a predictor for the odds to revert from chronic migraine to episodic migraine

	*Overall allodynia*			*Spatial distribution*			*Type of stimulus*		
	Multivariate OR (95% CI)		*p*	Multivariate OR (95% CI)		*p*	Multivariate OR (95% CI)		*p*
No allodynia									
*versus* allodynia	**2.45**	**(1.03; 5.84)**	**0.042**						
No allodynia									
*versus* cephalic allodynia				**4.16**	**(1.21; 14.30)**	**0.024**			
*versus* extracephalic allodynia				**7.32**	**(1.98; 27.11)**	**0.003**			
No allodynia									
*versus* thermal allodynia only							2.38	(0.47; 12.05)	0.297
*versus* mechanical + thermal allodynia							**5.61**	**(1.63; 19.30)**	**0.006**
*versus* mechanical allodynia only							7.52	**(1.60; 35.39)**	0.011

Adjusted for: gender, age, depression and anxiety

Cutaneous allodynia and the subtypes based on spatial distribution and type of stimulus were also predictive for the migraine specific secondary outcome measures. Participants without allodynia had a more than 2 times higher odds on ≥ 50% response (defined as ≥ 50% reduction in monthly migraine days) (OR 2.28; 95% CI 1.01 to 5.16; *p* = 0.048) (Table S2). The absence of allodynia was also predictive for the absolute reduction in monthly migraine days (MMD), with a reduction of 9.4 versus 5.9 MMD in participants without allodynia versus participants with allodynia (difference 3.49, 95% CI 0.95 to 6.02, *p* = 0.007). Similar to the primary outcome, the predictive value was more pronounced when subdivided by spatial distribution, and mainly related to mechanical allodynia, not thermal allodynia (mean differences in Table 3). However, neither cutaneous allodynia in general, nor the subtypes of cutaneous allodynia were predictive for reduction in monthly headache days (MHD) (Table 3). All the associations on primary and secondary outcomes did not alter after adjusting for medication days and migraine (MMD) or headache days (MHD) at baseline in supplementary analyses (data not shown).

**Table 3:** Allodynia as a predictor for reduction in monthly migraine days (MMD) and monthly headache days (MHD)

	*Overall allodynia*			*Spatial distribution*			*Type of stimulus*		
	Difference (95% CI)		*p*	Difference (95% CI)		*p*	Difference (95% CI)		*p*
**Reduction in MMD**									
Allodynia (no allodynia vs allodynia)	**3.49**	**(0.95; 6.02)**	**0.007**						
No allodynia									
*versus* cephalic allodynia				**3.35**	**(0.24; 6.45)**	**0.035**			
*versus* extracephalic allodynia				**4.96**	**(1.60; 8.32)**	**0.004**			
No allodynia									
*versus* thermal allodynia only							2.02	(-2.19; 6.23)	0.348
*versus* mechanical + thermal allodynia							**4.17**	**(1.06; 7.27)**	**0.009**
*versus* mechanical allodynia only							**5.22**	**(0.88; 9.55)**	**0.018**
**Reduction in MHD**									
Allodynia (no allodynia vs allodynia)	1.30	(-1.00; 3.59)	0.267						
No allodynia									
*versus* cephalic allodynia				2.09	(-0.74; 4.91)	0.148			
*versus* extracephalic allodynia				2.62	(-0.43; 5.66)	0.093			
No allodynia									
*versus* thermal allodynia only							2.57	(-1.24; 6.38)	0.187
*versus* mechanical + thermal allodynia							2.15	(-0.67; 4.97)	0.135
*versus* mechanical allodynia only							2.61	(-1.32; 6.54)	0.192

*MHD* Monthly headache days, *MMD* Monthly migraine days

Adjusted for: gender, age, depression and anxiety

In our secondary analyses, in addition to correcting for gender, age, depression and anxiety, we also corrected for whether patients received BTA treatment or placebo. Adding botulinum toxin A did not have major effects on the associations between (subtypes of) cutaneous allodynia and migraine-related outcome (Tables S3 and S4). For the primary endpoint, the odds for reversion from chronic migraine to episodic migraine was 2.4 times higher for participants without allodynia compared to participants with allodynia (OR 2.37; 95% CI 0.99 to 5.64; *p* = 0.053 (Table S3), only just not reaching statistical significance). The predictive value remained more pronounced when allodynia was specified according to spatial distribution (no allodynia versus cephalic allodynia OR 4.06; 95% CI 1.17 to 14.00; *p* = 0.027, no allodynia versus extracephalic allodynia OR 7.05; 95% CI 1.89 to 26.31, *p* = 0.004). When subdivided by type of stimulus, both the combination of mechanical plus thermal allodynia and mechanical allodynia alone were predictive for reversion to episodic migraine, whereas thermal allodynia alone was not predictive (Table S3). Cutaneous allodynia and the subtypes based on spatial distribution and type of stimulus were also still predictive for the migraine specific secondary outcome measures when BTA treatment was added to the model (Table S4).

## Discussion

This study shows that the absence of cutaneous allodynia is predictive for a good outcome after withdrawal therapy in patients with chronic migraine with medication overuse. The predictive value was even more pronounced when comparing with extracephalic allodynia, which is indicative of trigeminothalamic involvement. Our findings further suggest a migraine specific relationship because allodynia was only a strong predictor for migraine-related outcome measures.

These findings are relevant to clinical practice and current treatment concepts and expectations. Chronic migraine is a highly disabling migraine variant, in which the majority of patients overuse acute headache medication [3, 4]. Withdrawal of acute medication results into reversion to episodic migraine in the majority, but not of all, patients. Previous studies at predictors for response to withdrawal treatment in mixed populations of patients with migraine or tension type headache with medication overuse, mainly showed the underlying primary headache type as predictive factor [19, 20]. Daily headache or daily use of medication was a predictor in univariate analysis [19, 21], but did not predict outcome when adjusted for covariates [19]. Psychological factors have been indicated as predictor for response [20, 22], but require extensive assessment. This is the first study to show cutaneous allodynia as a predictor of response in chronic migraine, using a simple validated diagnostic tool for clinical practice. The effect size is moderate when comparing absence of allodynia versus allodynia in general (cohen’s d = 0.42), but increases to a moderate-large effect when considering spatial distribution, comparing no allodynia versus extracephalic allodynia (cohen’s d = 0.65). Especially with high-cost treatment with antibodies to Calcitonin gene-related peptide (CGRP) or its receptor [23, 24], identification of predictors for response to treatment is warranted. Various trials in chronic migraine demonstrated, partly in sub-analyses, that chronic migraine patients with medication overuse will be able to respond [25, 26]. However, no reliable predictors for response to monoclonal antibodies against CGRP or its receptor have yet been established. It is of great interest to investigate whether allodynia provides a specific predictor to chronic migraine and withdrawal therapy, or relates to other treatments in chronic migraine (with and without medication overuse) as well. In a post-hoc analysis of a trial of erenumab for treatment of chronic migraine (with and without medication overuse), erenumab led to similar improvements in patients with moderate-to-severe ictal allodynia and in those without ictal allodynia [27]. Importantly, the results of this trial could be influenced by i) a cohort in which both patient with and without medication overuse were included, ii) the absence of evaluating extracephalic allodynia and cephalic allodynia separately and ii) the fact that the majority of patients had no or minimal symptoms of allodynia. In contrast, more than 70% of migraine patients assessed with quantitative sensory testing had allodynia [6], and in our cohort ∼75% reported allodynia. As sustained exposure to acute headache medication in animal models causes allodynia and an increased sensitivity to cortical spreading depolarization, the associated increased CGRP release may mediate central sensitisation, thus leading to allodynia [5, 15, 28, 29]. Therefore, it seems prudent to investigate if allodynia may also be a predictor of response to anti-CGRP(-receptor) monoclonal antibody treatment in chronic migraine with medication overuse. Steps towards validating this hypothesis are currently being made. One study using real life data tried to identify predicters of response to anti-CGRP(-receptor) monoclonal antibodies treatment in patients with high frequent migraine attacks or chronic migraine [30], and allodynia was identified as a possible predictor. However, in contrast to our study, no validated questionnaire was used, a mix of patients with and without medication overuse were included, and no distinction was made between cephalic and extracephalic allodynia or spatial distribution. Interestingly, another study used Quantitative Sensory Testing (QST) to determine whether allodynia may predict response in chronic migraine and high frequent episodic migraine [31]. However, it is important to note that patients with medication overuse were excluded from that study in contrast to our study that only included patients with chronic migraine and medication overuse. Nonetheless, there findings may indicate that allodynia might be used to distinguish responders from non-responders prior to treatment with galcanezumab. These additional studies, indicate the importance of this line of research and underline that our findings are not necessarily related to withdrawal therapy.

The association between cutaneous allodynia, and its spatial distribution, and response to treatment may have additional value for current pathophysiological concepts on migraine chronification. The predictive value for failure on treatment was most pronounced for extracephalic allodynia, which is considered indicative for thalamic involvement [5–7]. Therefore, we hypothesize that especially thalamic involvement will be a predictor for unresponsiveness to treatment in chronic migraine patients. Until now, cutaneous allodynia has mainly been studied as a predictor of response to acute treatment with triptans or non-migraine specific acute headache medication, yielding contradictory results. Studies suggest that patients are unresponsive to triptans once cutaneous allodynia has manifested [32, 33]. While other studies suggest a preserved triptan response despite of cutaneous allodynia, as such the role of cutaneous allodynia on triptan response is not conclusive [34, 35]. However, while potentially important, the distinction between ipsi- and contra-lateral cephalic and/or extracephalic allodynia is not always made. The unresponsive to triptans once cutaneous allodynia has manifested led to the hypothesis that response to triptans may be indicative for different underlying sensitization mechanisms [6, 7, 34, 36]. Early in the sensitization process, when this mechanism is still depended on peripheral nociceptors, treatment appears to be more effective than during late sensitization, when there is no longer an influence of peripheral input. As triptans mainly appear to act peripherally [37], we can hypothesize that triptan-response occurs mainly while central sensitization is still developing and that it would cease upon thalamic involvement. Our study indicates that when late central sensitization has developed this will complicate potential preventive migraine treatment.

In our study, mechanical allodynia was associated with change in monthly migraine days, as opposed to thermal allodynia. This finding appears to be not due to a lack of power in the thermal allodynia group as both sample sizes were equal. So while we cannot exclude that with more power their might be a difference as well for thermal allodynia, these findings would suggest that mainly mechanoreceptors, such as the low threshold Aβ fibres and C-type mechanoreceptors [11, 36, 38], may be involved in migraine chronification. Although thermal allodynia is present in migraineurs as well during attacks, and in lesser extent in between attacks [6, 39], heat pain thresholds were not related to headache frequency [39], supporting our findings. This also fits with our conclusion that the predictive association was only present for migraine-related outcomes and not for headache days in general. In line with other studies, this suggests that central sensitization is more pronounced in migraine and not in other types of headaches [12]. Concordantly, a recent study investigating the ability to trigger cutaneous allodynia after nitroglycerine provocation, did not find an association between headache frequency and the occurrence of allodynia after nitroglycerine [34], whereas migraine frequency and occurrence of (spontaneous) cutaneous allodynia during migraine are shown to be related [9].

Strengths of this study are the large well-defined, representative chronic migraine population, with a high follow-up rate after withdrawal therapy and detailed information on headache characteristics, allodynia and psychiatric comorbidity. Due to detailed and prospective headache diaries, a distinction in migraine days and headache days could be made. The division in subtypes of cutaneous allodynia have never been studied related to chronic migraine in a longitudinal design. However, the subdivision on spatial distribution also has limitations. The Allodynia Symptom Checklist does not discern ipsilateral cephalic allodynia (second order neurons) and contralateral cephalic allodynia (third order neurons), as this cannot be reliably assessed in a questionnaire. Due to the division into different subgroups and the limited number of symptoms in the questionnaire, we used the criterion of at least one symptoms present for each subcategory, and not two or more as for the overall allodynia scores. Furthermore, while providing interesting avenues that are worth exploring and potentially creating a better understanding of the pathophysiological mechanisms involved in allodynia, we need to consider that while consistent across different migraine outcomes, our findings on allodynia caused by specific types of stimuli are based on a limited amount of participants. It would be worthwhile to evaluate our findings in a larger cohort. Moreover, the Allodynia Symptom Checklist is a self-reporting questionnaire and as such subjected to subjectivity. As such our measurements might therefore not be as reliable as the physiological assessment with QST, especially in assessing thermal allodynia, and possibly to a lesser extent mechanical allodynia. Nevertheless, as QST requires specialized equipment, training, and testing, is time consuming and costly, our evaluation is by far more applicable in clinical practice. Additionally, the study was part of a clinical trial on the effect of botulinum toxin A versus placebo, and we cannot fully rule out potential influence of the trial on the results. However, botulinum toxin A did not have additional benefit over placebo on all outcome measures [16], and adjusting for botulinum toxin A treatment did not have major effects on the associations between (subtypes of) cutaneous allodynia and migraine-related outcome. Animal studies suggest a different immune-mediated pathway for male and female [40], which might explain the difference in prevalence of cutaneous allodynia in male and female chronic migraine patients. Nevertheless, the association between cutaneous allodynia and response was adjusted for gender, and remained unchanged when analysis was rerun in female patients only making immune mediated influences of injection very unlikely.

## Conclusion

This study shows that self-reported cutaneous allodynia, an important marker for central sensitization, can potentially be used as a predictor for response to withdrawal therapy in patient with chronic migraine and medication overuse. Allodynia might be an important predictor for treatment response in chronic migraine in general. Furthermore, considering subtypes of cutaneous allodynia, especially extracephalic allodynia and mechanical allodynia, might enhance the predictive value for migraine-related outcomes and may help to increase insight in the mechanisms of chronification in migraine.

### Supplementary Information

**Additional file 1: Table S1.** Participants with no allodynia vs participants with at least one symptom of the different types of allodynia.** Table S2.** Allodynia as a predictor for the odds on ≥50% response in migraine days, i.e. a reduction in monthly migraine days of 50% or more.** Table S3.** Allodynia as a predictor for the odds to revert from chronic migraine to episodic migraine.** Table S4. **Allodynia as a predictor for reduction in monthly migraine days (MMD) and monthly headache days (MHD).

## Data Availability

The data that support the findings of this study are available on reasonable request from the corresponding author.

## Abbreviations

ASC: Allodynia Symptom Checklist
CGRP: Calcitonin gene-related peptide
ICHD: International Classification of Headache Disorders
HADS: Hospital Anxiety and Depression scale
MHD: Monthly headache days
MMD: Monthly migraine days
QST: Quantitative Sensory Testing
BTA: Botulinum toxin A
