# Supplementary material for: Cutaneous allodynia as predictor for treatment response in chronic migraine: a cohort study
Source: J Headache Pain. 2023 Aug 30;24(1):118. doi: 10.1186/s10194-023-01651-9 (PMC10466691; doi:10.1186/s10194-023-01651-9)
Supplement: Supplementary file 1 — Additional file 1: Table S1. Participants with no allodynia vs participants with at least one symptom of the different types of allodynia. Table S2. Allodynia as a predictor for the odds on ≥50% response in migraine days, i.e. a reduction in monthly migraine days of 50% or more. Table S3. Allodynia as a predictor for the odds to revert from chronic migraine to episodic migraine. Table S4. Allodynia as a predictor for reduction in monthly migraine days (MMD) and monthly headache days (MHD). [file 10194_2023_1651_MOESM1_ESM.pdf]

Table S1. Participants with no allodynia vs participants with at least one symptom of the different types of allodynia.

**Outcome**	**No allodynia*** (n=27)	**Cephalic allodynia** (n=79)	**Extracephalic allodynia** (n=67)	**Thermal allodynia** (n=16)	**Mechanical allodynia** (n=16)	**Thermal + mechanical allodynia** (n=114)
Reversion CM to EM	23 (85.2%)	49 (62.0%)	35 (52.2%)	12 (75.0%)	8 (50.0%)	64 (56.1%)
50% reduction MMD	18 (66.7%)	37 (46.8%)	28 (41.8%)	7 (43.8%)	6 (37.5%)	52 (45.6%)

Values are n (%). CM: chronic migraine; EM: episodic migraine; MMD: monthly migraine days. * Patients without a single symptoms of allodynia on the Allodynia Symptom Checklist.

Table S2. Allodynia as a predictor for the odds on ≥50% response in migraine days, i.e. a reduction in monthly migraine days of 50% or more.

	Overall allodynia			*Spatial distribution*			*Type of stimulus*		
	Multivariate OR (95% CI)		*p*	Multivariate OR (95% CI)		*p*	Multivariate OR (95% CI)		*p*
No allodynia *versus* allodynia	**2.28**	**(1.01 ; 5.16)**	**0.048**						
No allodynia									
versus cephalic allodynia				**3.88**	**(1.33; 11.35)**	**0.013**			
versus extracephalic allodynia				**6.17**	**(1.85 ; 19.45)**	**0.002**			
No allodynia									
versus thermal allodynia only							**4.57**	**(1.12 ; 18.69)**	**0.035**
versus mechanical + thermal allodynia							**4.34**	**(1.49 ; 12.64)**	**0.007**
versus mechanical allodynia only							**6.47**	**(1.52 ; 27.55)**	**0.012**

Adjusted for: gender, age, depression and anxiety.

Table S3. Allodynia as a predictor for the odds to revert from chronic migraine to episodic migraine.

	Overall allodynia			*Spatial distribution*			*Type of stimulus*		
	Multivariate OR (95% CI)		*p*	Multivariate OR (95% CI)		*p*	Multivariate OR (95% CI)		*p*
No allodynia *versus* allodynia	2.37	(0.99 ; 5.64)	0.053						
No allodynia									
versus cephalic allodynia				**4.06**	**(1.17; 14.00)**	**0.027**			
versus extracephalic allodynia				**7.05**	**(1.89 ; 26.31)**	**0.004**			
No allodynia									
versus thermal allodynia only							2.36	(0.47 ; 11.94)	0.299
versus mechanical + thermal allodynia							**5.45**	**(1.57 ; 18.88)**	**0.008**
versus mechanical allodynia only							**7.18**	**(1.50 ; 34.26)**	**0.013**

Adjusted for: gender, age, botulinum toxin A injections, depression and anxiety.

Table S4. Allodynia as a predictor for reduction in monthly migraine days (MMD) and monthly headache days (MHD).

	Overall allodynia			*Spatial distribution*			*Type of stimulus*		
	Difference (95% CI)		*p*	Difference (95% CI)		*p*	Difference (95% CI)		*p*
**Reduction in MMD**									
Allodynia (no allodynia vs allodynia)	**3.63**	**(1.08 ; 6.18)**	**0.005**						
No allodynia									
versus cephalic allodynia				**3.46**	**(0.35 ; 6.58)**	**0.029**			
versus extracephalic allodynia				**5.14**	**(1.76 ; 8.52)**	**0.003**			
No allodynia									
versus thermal allodynia only							2.09	(-2.12 ; 6.29)	0.331
versus mechanical + thermal allodynia							**4.31**	**(1.19 ; 7.43)**	**0.007**
versus mechanical allodynia only							**5.46**	**(1.10 ; 9.83)**	**0.014**
**Reduction in MHD**									
Allodynia (no allodynia vs allodynia)	1.12	(-1.19 ; 3.43)	0.34						
No allodynia									
versus cephalic allodynia				1.95	(-0.87 ; 4.78)	0.176			
versus extracephalic allodynia				2.41	(-0.66 ; 5.47)	0.124			
No allodynia									
versus thermal allodynia only							2.49	(-1.31 ; 6.29)	0.200
versus mechanical + thermal allodynia							1.98	(-0.85 ; 4.81)	0.169
versus mechanical allodynia only							2.33	(-1.62 ; 6.28)	0.248

MHD: monthly headache days; MMD: monthly migraine days. Adjusted for: gender, age, botulinum toxin A injections, depression and anxiety.
